# Sleep, Distressed Appearance, and Quality of Life Relate to Satisfaction with Orthognathic Surgery

**DOI:** 10.3390/ijerph182111253

**Published:** 2021-10-26

**Authors:** Yu-Shu Huang, Wei-Chih Chin, Chuan-Fong Yao, Ying-An Chen, I Tang, Yu-Ray Chen, Cheng-Hui Lin

**Affiliations:** 1 Department of Child Psychiatry and Sleep Center, Chang Gung Memorial Hospital and College of Medicine, Taoyuan 333, Taiwan; yushuhuang1212@gmail.com (Y.-S.H.); auaug0327@cgmh.org.tw (W.-C.C.); kikitang721@gmail.com (I.T.); 2 Department of Craniofacial Research Center, Chang Gung Memorial Hospital and College of Medicine, Taoyuan 333, Taiwan; chuanfongyao@gmail.com (C.-F.Y.); whysomimi@gmail.com (Y.-A.C.); 3 Department of Clinical Psychology, Fu Jen Catholic University, New Taipei City 24205, Taiwan

**Keywords:** orthognathic surgery, patient satisfaction, risk factors, quality of life, sleep

## Abstract

Background: In this study, we aimed to identify factors correlating with satisfaction with orthognathic surgery in order to improve its outcome. Methods: We recruited 77 participants who had received orthognathic surgery and 32 age- and gender-matched normal-controls. Questionnaires that included devised questions for family support, Big Five Inventory, Derriford Appearance Score, Pittsburgh Sleep Quality Index, Hospital Anxiety and Depression Scale, 36-Item Short-Form Health Survey, and a visual analogy scale for satisfaction, were completed before and one month and nine months after the surgery. The statistical analysis methods included descriptive statistics, *t*-test, and Pearson correlation. Results: All participants received the preoperative and one-month follow-up, while 28 also completed the nine-month follow-up. Satisfaction was not significantly related to demographic data, but long-term satisfaction was related to an extraverted personality. The preoperative and postoperative results of the Derriford Appearance Scale were related to short-term and long-term satisfaction. Furthermore, both the preoperative and one-month postoperative Pittsburgh Sleep Quality Index findings were significantly related to short-term satisfaction. The postoperative 36-Item Short-Form Health Survey was significantly related to short-term and long-term satisfaction. Conclusions: Not only subjective distress and dysfunction of appearance but also sleep problems and quality of life were correlated to satisfaction with orthognathic surgery. In the future, relevant interventions can be developed to further improve patient’s satisfaction and their physical and mental health.

## 1. Introduction

The purpose of orthognathic surgery (OGS) is both functional and aesthetic. Correction of malocclusion, which is defined as poor dental and jaw relationship, can lead to improved bite and a more pleasing appearance in those with asymmetrical faces. Studies have shown that patients with class II and III malocclusion experience more distress and insecurity compared to the control group regarding their facial appearance [1,2,3], while class III patients exhibit more psychological stress in social situations than those with other jaw deformities [1]. The benefits of OGS with regard to patients’ improved quality of life have been extensively reviewed [4,5,6], and the positive effects of OGS on psychosocial status have also been well reported [6,7,8].

However, some patients still report dissatisfaction with their outcome, despite objective improvement of skeletal imaging, orthodontic and surgical assessment, and the subjective improvement of appearance according to people around them [5,9,10,11]. Such unsatisfied patients can make multiple clinic visits, and some even insist on repeating the operation, which requires more time and unexpected costs for the medical team.

The exact reasons why some patients report improved quality of life and satisfying outcomes while others do not are still unknown [12]. Several factors may explain the differences in satisfaction. Psychological issues such as chronic insomnia or personality disorders can play a part. Patients with such conditions can have an increased risk of postoperative dissatisfaction if their psychological needs are unnoticed or unmanaged before surgery [13]. One study has also reported that patients with more psychological distress prior to surgery tend to experience more difficulties and discomfort after surgery [14]. Therefore, despite best efforts and surgical success, patients may remain unhappy and unsatisfied. Poor patient preparation for the surgery can be another related factor. Patients with unrealistic expectations of the surgery may be more likely to feel unsatisfied with the surgery, when the postoperative pain and discomfort is unexpected. Anticipatory anxiety preoperatively increases patients’ distress, and patients may adjust poorly to their new facial appearance postoperatively. All of these poorly prepared mental conditions can impact patients’ satisfaction. A previous study revealed that pre-surgery preparation increased the success rate of the surgery [14].

Medical teams are unable to detect potential psychosocial concerns of patients without a comprehensive screening protocol for baseline mental status before OGS. Since no postoperative psychosocial evaluation is provided to patients receiving OGS, no psychosocial intervention has been developed to improve surgical outcome and satisfaction. Therefore, identifying the factors related to satisfaction is crucial in order to optimize patient management strategies for OGS. We have previously reported on both the short-term and the long-term psychological impact and quality of life of patients undergoing orthognathic surgery [15]. In this study, we further analyzed the data of our prospective follow-up study and aimed to identify related psychosocial factors in patients undergoing surgery. We analyzed the correlation between these factors and short-term (one month after surgery) and long-term (nine months after surgery) satisfaction of OGS.

## 2. Materials and Methods

We prospectively recruited 77 patients who would receive OGS. Patients with a diagnosis of class II (*n* = 15, 19.5%) or III (*n* = 58, 75.3%) malocclusion and asymmetrical face (*n* = 4, 5.2%) were included, but patients with craniofacial syndrome, cleft lip and palate, facial deformities secondary to trauma, or tumor resections were excluded. We also recruited 32 gender- and age-matched normal healthy controls as the control group.

Recruited participants were scheduled for OGS at the Craniofacial Center in Chang Gung Memorial Hospital. We explained the purpose and process of the study to patients and their families, and all participants signed informed consent. We arranged psychological screening and evaluation assessment before the surgery, and participants completed questionnaires during this pre-surgery phase. In general, the recovery time for soft tissue is about three months, and it takes about six months for bone tissue stabilization and accommodation following OGS, although some patients may need longer. Therefore, after the surgery, we followed up these participants, who then completed the same questionnaires one month after surgery (short-term follow-up) and nine months after surgery (long-term follow-up). The control group only received the baseline assessment. This study was approved by the institutional review board of Chang Gung Memorial Hospital (No. 201600881B0), and all patients signed informed consent to participate in the study.

### 2.1. The Surgical Technique of OGS

Orthognathic surgery (OGS) is a safe and essential surgery for functional correction of malocclusion and to esthetically improve facial profile [16,17]. It is recommended for patients with craniofacial anomaly, acquired dentofacial deformity, or facial asymmetry [18]. OGS mainly consists of two key surgical techniques including Le Fort I osteotomy (LFI) and bilateral sagittal splitting osteotomy (BSSO). LFI is used to disjoin the connection of the maxilla to the pterygoid plate and zygoma to free the upper jaw. Meanwhile, BSSO can separate the proximal and distal segments of the mandible to free the lower jaw. Then, based on the guided stent, a new maxillomandibular complex could be repositioned for a better facial profile according to intra-operative esthetic checkpoints [19]. Finally, genioplasty is usually performed after either jaw surgery to optimize facial harmony.

### 2.2. Psychological and Questionnaire Assessment

We included various questionnaires in order to comprehensively assess our participants. The psychological screening and evaluation assessment addressed the following six domains: (1) Demographic data, including family support, (2) Personality assessment, (3) Measurement of distress and dysfunction regarding problems of appearance, (4) Quality of sleep, (5) Emotional assessment, and (6) Quality of life. Satisfaction with the surgery was also evaluated.

- Demographic data and family support: Demographic data such as gender, age, and physical/psychological conditions were collected. Family support was evaluated by our devised questions regarding the education level of parents, the attitude of the family to the surgery, and the family’s relationship to the clinic. Previous studies have reported well-established links between family and social support to physiological behavior and health [20].
- Personality assessment: The Big Five Inventory (BFI) has 33 major questions assessing five personality components, including extraversion, agreeableness, conscientiousness, neuroticism, and openness. Higher scores indicate that a patient is more likely to have a specific personality trait [21].
- Measurement of distress and dysfunction regarding problems of appearance: The Derriford Appearance Score (DAS-24) is a scale measuring distress and dysfunction of appearance and can be applied to different causes of appearance-altering conditions, such as burns, cleft lip and palate, jaw deformities, etc. [22,23].
- Quality of sleep: The Pittsburgh Sleep Quality Index (PSQI) Scale has nine major questions that assess eight sleep components. These components include subjective sleep quality, sleep latency, sleep duration, habitual sleep efficiency, sleep disturbances, use of sleeping medication, daytime dysfunction, and global PSQI score [24]. Higher scores represent worse sleep quality.
- Emotional assessment: The Hospital Anxiety and Depression Scale (HADS) includes 14 major questions assessing two components: depression and anxiety. Higher scores mean more depression or anxiety. The Chinese-Cantonese HADS has demonstrated internal consistency. Cronbach’s alpha is 0.86 for the full scale, 0.82 for the depression subscale, and 0.77 for the anxiety subscale [25].
- Quality of life: The 36-Item Short-Form Health Survey (SF-36) includes 11 major questions assessing eight components, including physical functioning, role limitations due to physical health, role limitations due to emotional problems, energy/fatigue, emotional well-being, social functioning, pain, and general health. The scale can be used to estimate a patient’s general status of quality of life. Higher scores indicate better quality of life [26].
- Satisfaction: We designed a visual analogy scale scored from 1 to 10. The score “10” indicates the most satisfaction, “1” is the most dissatisfaction, and “5” is moderate satisfaction. Participants provided scores at one month and nine months after OGS to evaluate short-term and long-term satisfaction.

### 2.3. Statistics

We use SPSS, version 20 (SPSS, Inc., Chicago, IL, USA) to analyze these data. Variables were presented as either mean ± standard deviation or frequency. We used Chi-square test or *t*-test to evaluate pre- and post-surgery data. The correlation was performed by Pearson’s correlation coefficient analysis. A *p*-value of less than 0.05 was considered statistically significant.

## 3. Results

We enrolled a total of 77 participants (male = 32.5%, mean age = 22.36 ± 7.97 years) receiving OGS in the OGS group, who completed the assessment before the surgery and the follow-up assessment one month after the surgery. The control group consisted of 32 age- and gender-matched normal healthy controls (male = 40.6%, mean age = 21.78 ± 6.69 years). Only 28 participants in the OGS group also completed the nine-month follow-up. Table 1 shows that the percentage of the most satisfaction was more than 70% both at one month and nine months after OGS (78.6% and 70.6%), and the least satisfaction was 7.1% and 5.9% at one month and nine months after OGS, respectively. Furthermore, satisfaction was not significantly related to any demographic data or family support. Table 2 shows the correlation between BFI and HADS and surgical satisfaction. BFI and HADS did not indicate a significant correlation with satisfaction one month or nine months after OGS, except that nine-month postoperative extraversion was positively related with satisfaction nine months after OGS (*r* = 0.531, *p* = 0.009).

Table 3 shows that preoperative distress at reflection (*r* = −0.419, *p* = 0.037), consciousness of appearance (*r* = −0.403, *p* = 0.045), and one-month postoperative irritability at home ((*r* = −0.507, *p* = 0.011) were related to satisfaction one month after OGS. Nine-month postoperative distress at reflection (*r* = −0.687, *p* = 0.010), irritability at home (*r* = −0.662, *p* = 0.014), feeling hurt (*r* = −0.707, *p* = 0.007), self-conscious of appearance (*r* = −0.633, *p* = 0.020), feeling irritable (*r* = −0.808, *p* = 0.001), distress at social events (*r* = −0.580, *p* = 0.038), and feeling normal (*r* = −0.594, *p* = 0.032) were all related to satisfaction nine months after OGS.

Sleep problems evaluated by the PSQI showed that both preoperative and one-month postoperative subjective sleep quality (*r* = −0.454, *p* = 0.023; *r* = −0.455, *p* = 0.022) and sleep disturbances (*r* = −0.464, *p* = 0.022; *r* = −0.415, *p* = 0.039) were related to satisfaction one month after OGS, as well as one-month postoperative global PSQI score (*r* = −0.410, *p* = 0.046) (Table 4). We observed no significant correlation between PSQI and satisfaction nine months after OGS, indicating that sleep problems negatively correlated only with short-term satisfaction.

Quality of life evaluated by SF-36 showed that one-month postoperative role limitations due to physical health (*r* = 0.449, *p* = 0.031) and body pain (*r* = 0.499, *p* = 0.015) were related to satisfaction one month after OGS (Table 5). Nine-month postoperative role limitation due to physical health (*r* = 0.423, *p* = 0.045), energy/fatigue (*r* = 0.446, *p* = 0.033), and mental health (*r* = 0.513, *p* = 0.012) were related with satisfaction nine months after OGS.

## 4. Discussion

In this prospective case-control study with long-term follow-up, we used different statistical methods to determine factors that could impact the outcome and satisfaction of OGS. This study had some limitations. First, the sample size was not large, and more than half of our participants dropped out from the study nine months after OGS. The low return rate may relate to differences in healthcare-seeking behavior, and further studies with more participants are needed to explore this issue. Second, we could not analyze the differences between satisfied and unsatisfied participants, since the sample size of the dissatisfaction group was too small due to overall high satisfaction, and limited to the sample size and study design, we could not analyze the differences between subgroups, such as patients with different purposes for OGS. Third, we did not use objective measurements to quantify sleep, such as polysomnography or actigraphy. As a result, sleep disorders such as obstructive sleep apnea could not be ruled out, and patients with specific sleep disorders may have had different clinical outcomes. Fourth, though with overall high satisfaction, body dysmorphic disorder was not screened in this study, and these patients could be unsatisfied with OGS. Nevertheless, with only subjective measurements, our psychological assessment was comprehensive, and the follow-up was up to nine months after the surgery.

As for the results of this study, both short-term and long-term postoperative satisfaction with OGS was high. Demographic data including gender, age, education of patients, and physical conditions did not significantly relate to satisfaction. Psychological conditions, family support, personality, and emotion assessment by BFI and HADS also did not show significant correlations, except that extroverted personality nine months after OGS was positively related to long-term satisfaction. Previous studies have shown that negative predictors of post-operative satisfaction have been linked to personality traits such as having an obsessive personality or narcissistic personality [27,28,29,30]. Depression also plays a role in surgical outcome, and patients with depression had less improvement in quality of life after OGS [12]. Another study investigating the impact of social support on surgical satisfaction of OGS also reported a positive correlation [31]. These results should be interpreted with caution and may be explained by differences in study populations. Our OGS patients were relatively young, mostly young adults or adolescents, with fair family support. They did not show significant differences in BFI or HADS compared with the control group (the results were shown in another of our studies under review), and most of them did not have a psychological condition or specific personality disorder. The satisfaction of these patients without comorbid mental illness was generally good, and the correlation between family support, personality, mental illness, and surgical satisfaction may be much lower. BFI and HADS can serve as good screening tools for assessing personality and psychological factors prior to OGS.

Our previous findings of DAS-24 revealed significant improvements that persisted to nine months after OGS [15], and we further revealed that distress and dysfunction regarding problems of appearance assessed with DAS-19 were significantly related to surgical satisfaction in this study. Preoperative and one-month postoperative distress negatively correlated with short-term satisfaction, and nine-month postoperative distress negatively correlated with long-term satisfaction. The fact that distress regarding appearance had more long-term impacts on satisfaction warrants close and long-term follow-up. Although these findings of DAS-24 are not surprising, we should also note that not all items of the scale related to satisfaction. In particular, those with significant correlation are often related to social and emotional distress, and more social anxiety has been reported in orthognathic patients [32]. Our BFI results also supported that if patients became more extroverted after OGS, they were more satisfied, indicating the possible role of socialization and its postoperative impact on these patients. Therefore, although surgical success remains the most important aspect of OGS, interventions targeting self-image, emotion management, and interpersonal skills can be developed in order to further assist these patients.

One interesting finding involves patients’ sleep. We found that preoperative and one-month postoperative sleep quality and sleep disturbances could influence short-term satisfaction, and one-month postoperative global PSQI score also influenced short-term satisfaction. The sleep of patients receiving OGS can fluctuate. Sleep latency was found to be exacerbated after OGS and improved nine months later [15]. Sleep deprivation not only increases physiological reactivity to stressors but also reduces the psychological threshold for stress management [33,34]. Although long-term satisfaction did not reveal a significant correlation with sleep, a brain imaging study concluded that subjective sleep from only the preceding night could be negatively correlated with prefrontal–amygdala connectivity and the severity of subjective psychological distress [35]. Therefore, sleep should be monitored preoperatively and at least short-term after OGS. Triggers for poor sleep should be identified, such as anticipatory anxiety before OGS or wound pain after OGS, and then managed as needed.

Last, we found that some domains of SF-36 also influenced satisfaction. One-month postoperative body pain and role limitations due to physical health related to short-term satisfaction represent factors that can be noted clinically. Surgical skills and anesthetic protocols chosen during surgery can both influence postoperative pain, as well as post-surgery care and pain control [14]. One study showed that steroids could reduce the symptoms of swelling, pain, nausea, and vomiting and increase nerve healing after OGS [14]. Moreover, short-term satisfaction correlated only with the physical domains of SF-36, while long-term satisfaction correlated with both physical and psychological domains of SF-36, including role limitations due to physical health, energy/fatigue, and mental health. While attention should be paid to post surgery care and pain control early after OGS, surgeons should also be aware of other long-term aspects. Nine months after the surgery, role limitation due to physical health can still be worse than healthy controls even with high satisfaction of OGS [15]. A longitudinal study investigating psychosocial changes throughout OGS showed that 15% of patients could be chronically dysfunctional and distressed, even after OGS [36]. Interventions such as exercise to generate energy and mental support to decrease stress should be provided and encouraged. Some patients may even need referrals to such specialties as psychiatrists or rehabilitation centers for further treatment.

## 5. Conclusions

With overall fair satisfaction after OGS, our study suggested that not only the subjective distress and dysfunction of appearance but also sleep problems and quality of life correlated to satisfaction with OGS. Although surgical success is still the main concern, preoperative and postoperative assessments of these factors are important. DAS-24 and PSQI are effective candidate questionnaires for evaluating the psychological conditions of patients receiving OGS, and SF-36 can be used to evaluate their quality of life. Interventions targeting these satisfaction-related variables, such as treatment of sleep problems, pain control, emotion management, and social skill training, can be developed to further improve satisfaction, as well as both the physical and mental health of patients receiving OGS. Furthermore, studies with longer follow-up periods can help confirm our findings. By using different measurements, such as polysomnography and psychiatric interview or psychological tests, specific groups of patients, such as those with personality disorder, depression, anxiety, obstructive sleep apnea, or insomnia, can be targeted to identify factors related to the satisfaction of OGS. Other issues worthy of further study include different healthcare-seeking behaviors and cultural views on aesthetics and OGS, as well as different purposes of OGS such as cosmetic, biting or sleep disturbances, which can relate to dropout rate and impact satisfaction.

## Figures and Tables

**Table 1:** Surgical satisfaction of OGS and its correlation with demographic data and family support.

	Satisfaction (1 Month after OGS) (N = 77)				Satisfaction (9 Months after OGS) (N = 28)			
	Male (32.5%)	Female (67.5%)	Total	*p*	Male (39.3%)	Female (60.7%)	Total	*p*
VAS of Satisfaction (M ± SD)	8.50 ± 1.65	8.56 ± 1.50	8.54 ± 1.79	0.981	8.17 ± 1.84	8.60 ± 1.35	8.70 ± 1.25	0.875
VAS: 1 to 5	10%	5.6%	7.1%		14.3%	0.0%	5.9%	
VAS: 6 to 7	10%	16.7%	14.3%		0.0%	30%	17.6%	
VAS: 8 to 10	80.0%	77.8%	78.6%	0.551	71.4%%	70.0%%	70.6%	0.891
	**Satisfaction** **(1 Month after OGS) (N = 77)**				**Satisfaction** **(9 Months after OGS) (N = 28)**			
Demographic data			*r*	*p*			*r*	*p*
Gender			−0.018	0.929			−0.144	0.595
Age			−0.030	0.880			0.231	0.390
Education of participants			0.015	0.944			−0.227	0.415
Physical conditions			−0.156	0.466			−0.379	0.181
Psychological conditions			0.089	0.679			−0.370	0.193
Family support								
Education of father			−0.034	0.874			−0.092	0.754
Education of mother			0.178	0.405			0.105	0.721
Does the family agree?			0.234	0.271			0.145	0.621
Accompanied by relatives to the clinic			−0.146	0.507			0.202	0.489

*p* values were calculated using Chi-square test or *t*-test of two groups (male and female); OGS: orthognathic surgery; VAS: visual analogy scale (score from 1 to 10, with “10” meaning the most satisfied).

**Table 2:** Correlation between BFI and HADS (preoperative, 1 month and 9 months after OGS) and surgical satisfaction.

	Satisfaction (1 Month after OGS) (N = 77)				Satisfaction (9 Months after OGS) (N = 28)			
	Preoperative BFI and HADS		1 Month after Surgery BFI and HADS		Preoperative BFI and HADS		9 Months after Surgery BFI and HADS	
	*r*	*p*	*r*	*p*	*r*	*p*	*r*	*p*
BFI-Openness	0.225	0.280	0.282	0.171	−0.066	0.754	0.087	0.694
BFI-Conscientiousness	0.002	0.994	0.307	0.135	0.154	0.463	0.275	0.203
BFI-Extraversion	0.218	0.296	0.400	0.053	0.275	0.183	0.531	0.009 *
BFI-Agreeableness	0.029	0.892	0.316	0.124	0.265	0.200	0.270	0.212
BFI-Neuroticism	−0.274	0.185	−0.051	0.807	−0.151	0.472	0.018	0.934
HADS-Depression	−0.288	0.154	−0.063	0.775	−0.062	0.767	0.030	0.891
HADS-Anxiety	−0.278	0.169	−0.183	0.404	0.046	0.826	0.093	0.673

Pearson’s correlation coefficient (*r*) analysis. * *p*-value < 0.05. OGS: orthognathic surgery; BFI: Big Five Inventory; HADS: Hospital Anxiety and Depression Scale.

**Table 3:** Correlation between DAS-24 (preoperative, 1 month and 9 months after OGS) and surgical satisfaction.

	Satisfaction (1 Month after OGS 1 Month) (N = 77)				Satisfaction (9 Months after OGS) (N = 28)			
	Preoperative DAS-24		1 Month after OGS DAS-24		Preoperative DAS-24		9 Months after OGS DAS-24	
	*r*	*p*	*r*	*p*	*r*	*p*	*r*	*p*
Distress at reflection	−0.419	0.037 *	−0.316	0.132	0.000	10.000	−0.687	0.010 *
Irritable at home	−0.114	0.587	−0.507	0.011 *	−0.010	0.971	−0.662	0.014 *
Feeling hurt	−0.278	0.188	−0.191	0.371	−0.101	0.710	−0.707	0.007 *
Self-consciousness affects work	−0.116	0.582	0.067	0.755	0.034	0.902	−0.400	0.175
Distressed at beach	0.182	0.384	0.028	0.895	−0.224	0.403	−0.224	0.463
Misjudged due to appearance	−0.073	0.727	−0.199	0.351	0.082	0.763	−0.256	0.399
Self-conscious of appearance	−0.403	0.045 *	−0.209	0.338	−0.020	0.943	−0.633	0.020 *
Feeling irritable	−0.293	0.155	−0.297	0.158	0.094	0.728	−0.808	0.001 *
Adopt concealing gestures	−0.333	0.103	−0.106	0.621	0.152	0.574	0.217	0.477
Avoid communal changing	−0.235	0.270	−0.059	0.784	0.004	0.989	0.306	0.310
Distressed in supermarkets/department stores	−0.070	0.745	−0.369	0.076	0.082	0.770	−0.299	0.321
Avoid undressing with partner	0.049	0.819	−0.288	0.172	0.206	0.461	0.198	0.516
Distressed playing sport/games	−0.144	0.503	−0.037	0.865	−0.092	0.745	0.029	0.925
Distressed by clothing limitations	0.141	0.512	−0.101	0.638	0.227	0.416	0.224	0.461
Distressed at social events	−0.083	0.707	−0.150	0.485	0.117	0.690	−0.580	0.038 *
Feeling normal	0.008	0.972	−0.129	0.548	0.441	0.099	−0.594	0.032 *
Affects sex life	0.004	0.987	−0.211	0.322	0.155	0.581	0.392	0.185
Distressed at others remarks about appearance	−0.177	0.409	−0.120	0.576	−0.087	0.759	−0.356	0.233
Avoid pubs/restaurants	0.155	0.469	−0.248	0.254	0.065	0.819	0.095	0.758

Pearson’s correlation coefficient (*r*) analysis. * *p*-value < 0.05. OGS: orthognathic surgery; DAS: Short-form of the Derriford Appearance Scale.

**Table 4:** Correlation between the PSQI (preoperative, 1 month and 9 months after OGS) and surgical satisfaction.

	Satisfaction (1 Month after OGS) (N = 77)				Satisfaction (9 Months after OGS) (N = 28)			
	Preoperative PSQI		1 Month after OGS PSQI		Preoperative PSQI		9 Months after OGS PSQI	
	*r*	*p*	*r*	*p*	*r*	*p*	*r*	*p*
Subjective sleep quality	−0.454	0.023 *	−0.455	0.022 *	−0.054	0.796	0.119	0.589
Sleep latency	−0.256	0.217	−0.345	0.091	−0.146	0.485	−0.117	0.596
Sleep duration	−0.130	0.534	−0.295	0.153	0.159	0.448	−0.082	0.709
Habitual sleep efficiency	−0.164	0.443	−0.107	0.619	−0.120	0.567	−0.039	0.861
Sleep disturbances	−0.464	0.022 *	−0.415	0.039 *	−0.294	0.162	−0.084	0.705
Use of sleeping medication	0.059	0.780	0.118	0.573	−0.107	0.609	−0.003	0.988
Daytime dysfunction	−0.333	0.103	−0.268	0.195	−0.147	0.482	−0.348	0.104
Global PSQI Score	−0.376	0.067 ^+^	−0.410	0.046 *	−0.147	0.492	−0.133	0.547

Pearson’s correlation coefficient (*r*) analysis. * *p*-value < 0.05.; + *p*-value < 0.1. OGS: orthognathic surgery; PSQI: Pittsburgh Sleep Quality Index.

**Table 5:** Correlation between SF-36 (preoperative, 1 month and 9 months after OGS) and surgical satisfaction.

	Satisfaction (1 Month after OGS) (N = 77)				Satisfaction (9 Months after OGS) (N = 28)			
	Preoperative SF-36		1 Month after OGS SF-36		Preoperative SF-36		9 Months after OGS SF-36	
	*r*	*p*	*r*	*p*	*r*	*p*	*r*	*p*
Physical Function	−0.222	0.286	−0.151	0.493	0.193	0.355	−0.062	0.779
Role Limitations Due To Physical Health	−0.135	0.519	0.449	0.031 *	0.115	0.585	0.423	0.045 *
Role Limitations Due To Emotional Problems	0.085	0.686	0.249	0.252	0.020	0.924	0.295	0.172
Energy/Fatigue	0.356	0.080 ^+^	0.323	0.133	0.206	0.322	0.446	0.033 *
Mental Health	0.165	0.432	0.053	0.811	0.136	0.516	0.513	0.012 *
Social Functioning	0.171	0.413	0.325	0.130	0.169	0.419	0.102	0.643
Body Pain	0.224	0.283	0.499	0.015 *	0.089	0.671	−0.156	0.477
General Health	0.392	0.050 ^+^	0.193	0.378	0.210	0.314	−0.464	−0.062

Pearson’s correlation coefficient (*r*) analysis. * *p*-value < 0.05.; + *p*-value < 0.1. OGS: orthognathic surgery; SF-36: 36-Item Short-Form Health Survey.

## Data Availability

The data presented in this study are available from the corresponding author upon reasonable request.
